# The Future of Scientific Writing: AI Tools, Benefits, and Ethical Implications

**DOI:** 10.1590/0103-644020256471

**Published:** 2025-04-07

**Authors:** José Mauro Granjeiro, Altair Antoninha Del Bel Cury, Jaime Aparecido Cury, Mike Bueno, Manoel Damião Sousa-Neto, Carlos Estrela

**Affiliations:** 1 Division of Metrology in Biology, National Institute of Metrology, Quality and Technology, Duque de Caxias, RJ, Brazil.; 2 Department of Prosthodontics and Periodontology, Piracicaba Dental School, University of Campinas (UNICAMP), Piracicaba, SP, Brazil.; 3 Department of Biosciences, Piracicaba Dental School, University of Campinas (UNICAMP), Piracicaba, SP, Brazil.; 4 São Leopoldo Mandic University, Faculty of Dentistry, Department of Radiology, Campinas, SP, Brazil.; 5 Department of Restorative Dentistry, School of Dentistry of Ribeirão Preto, University of São Paulo (USP), Ribeirão Preto, SP, Brazil.; 6 Department of Stomatologic Science, Federal University of Goiás, Goiânia, GO, Brazil.

**Keywords:** Artificial intelligence, writing, dentistry, communication barriers, ethics

## Abstract

Artificial Intelligence (AI) transforms scientific writing by improving efficiency, accessibility, and quality. This study evaluated the applications, benefits, and challenges of AI tools, including Elicit, Perplexity, Consensus, ChatGPT, and Grammarly, in the literature review, information organization, and textual clarity enhancement. A narrative review and practical analysis were conducted, assessing the tools based on synthesis capabilities, accessibility, and accuracy. Results showed that AI tools optimize literature analysis and enhance the clarity of scientific texts, particularly for non-native English-speaking researchers. However, limitations include technical inaccuracies, excessive standardization of writing style, and ethical concerns regarding authorship and accountability. The study concludes that while AI can significantly support scientific writing, its adoption should be accompanied by stringent human oversight and adherence to ethical guidelines to maintain academic integrity.

## Resumo

A inteligência artificial (IA) está transformando a escrita científica, promovendo maior eficiência, acessibilidade e qualidade. Este estudo teve como objetivo avaliar as aplicações, benefícios e desafios de ferramentas de IA, como Elicit, Perplexity, Consensus, ChatGPT e Grammarly, na revisão de literatura, organização de informações e melhoria da clareza textual. Foi realizada uma revisão narrativa e uma análise prática das ferramentas, avaliando critérios como capacidade de síntese, acessibilidade e precisão. Os resultados demonstraram que as ferramentas de IA otimizam a análise de literatura e melhoram a clareza de textos científicos, especialmente para pesquisadores não nativos na língua inglesa. No entanto, foram identificadas limitações, como imprecisões técnicas, padronização excessiva do estilo de escrita e preocupações éticas relativas à autoria e à responsabilidade. Conclui-se que, embora a IA possa apoiar significativamente a escrita científica, sua adoção deve ser acompanhada de uma rigorosa supervisão humana e de diretrizes éticas para garantir a integridade acadêmica.

**Figure:**
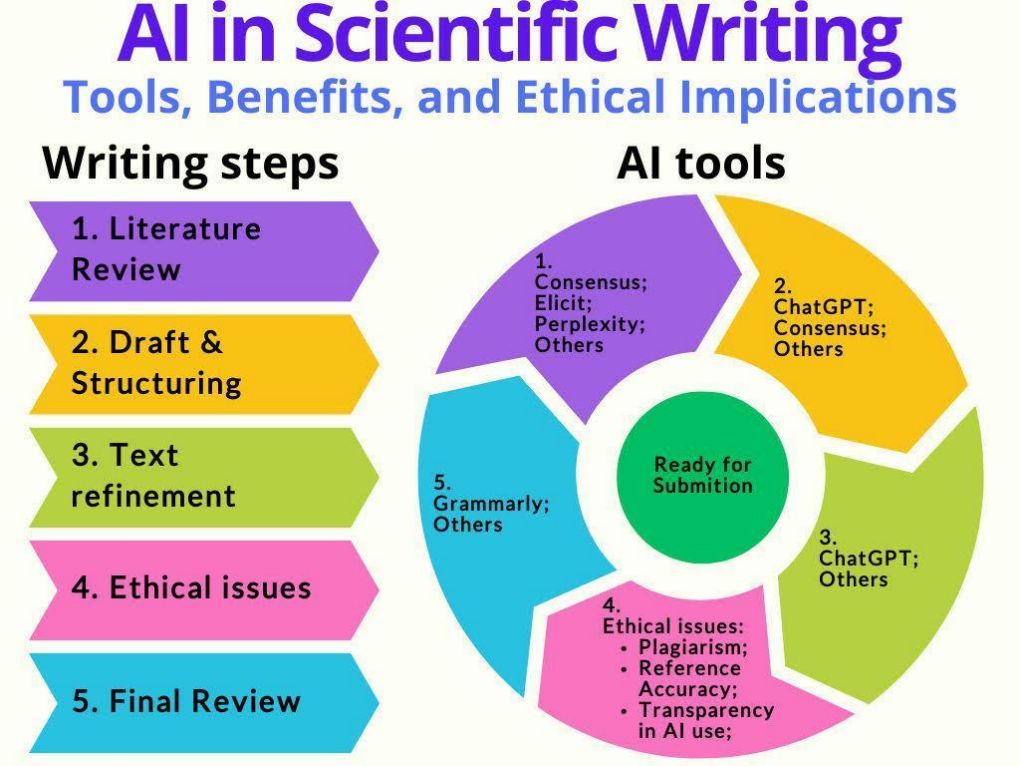

## Introduction

Artificial intelligence (AI) is reshaping how researchers communicate and share their findings, providing tools that improve the efficiency, accessibility, and reach of scientific writing. In academic research, where clarity and precision are essential for advancing knowledge and practice, AI-driven platforms like Perplexity, Consensus, and Elicit have become valuable resources. These technologies not only optimize the organization and synthesis of existing literature but also facilitate the identification of research gaps and the development of robust scientific hypotheses and research questions^1^.

The intelligent use of technology enables deeper analysis, fostering the generation of valuable insights that can accelerate innovation and critically assess the quality of scientific projects and writing^2^. With the support of these tools, researchers are equipped to refine their investigations, enhance clinical decision-making, and contribute to a more comprehensive and up-to-date body of knowledge within the field^3^. Furthermore, these AI-driven solutions enable more precise research methodologies and promote evidence-based practices that drive continuous advancements across various fields of knowledge.

For many researchers who are non-native English speakers, tools such as ChatGPT and Grammarly have become invaluable allies, helping to overcome language barriers by improving the clarity, coherence, and fluency of their texts, thus enhancing the visibility of their scientific findings^3^ ^,^ ^4^. By offering substantial benefits, these tools facilitate more effective communication in the global scientific community, enabling researchers to reach a wider audience and engage in scholarly discourse across linguistic borders^3^. However, despite these significant advantages, notable obstacles remain, such as the risk of excessive standardization of language and the potential for technical inaccuracies that could affect the integrity of scientific writing. These issues underscore the critical importance of human oversight and the need for well-defined ethical guidelines to ensure that the use of AI in scientific writing remains rigorous, reliable, and aligned with academic standards^5^ ^,^ ^6^.

This study aims to evaluate and highlight the applications of AI tools - such as Elicit, Perplexity, Consensus, ChatGPT, and Grammarly - in scientific writing, focusing on literature review, information organization, and textual clarity improvement within dentistry. Additionally, this article addresses these technologies' limitations and ethical challenges.

## Materials and methods

This study employed a narrative analysis based on a non-systematic literature review, complemented by the author's experience in using artificial intelligence (AI) tools for scientific writing. The bibliographic search was conducted using PubMed, Scopus, and Web of Science, with the terms "artificial intelligence" and "scientific writing". Articles published within the last five years (2019-2024) were prioritized, focusing on the benefits, limitations, and ethical challenges associated with using AI.

The evaluation of AI tools followed specific stages, using criteria such as synthesis capability, accessibility, and information accuracy. Elicit was assessed for its efficiency in generating article summaries based on targeted queries. Its metadata filtering features and integration with reference management software, such as Zotero and Mendeley, were tested in simulated bibliographic reviews. Perplexity was evaluated for its performance in retrieving recent information and generating concise summaries with contextual citations. Meanwhile, Consensus was tested for its ability to identify research gaps and provide evidence-based summaries. This approach aimed to assess the potential of these technologies to facilitate the communication of research findings and to identify gaps in the scientific literature within the field of dentistry^7^ ^,^ ^8^.

In addition to literature tools, ChatGPT and Grammarly were applied in practical scientific writing exercises. ChatGPT was evaluated for its capacity to generate drafts, suggest research topics, and synthesize findings. Grammarly reviewed text, focusing on grammar correction, improving textual cohesion, and detecting plagiarism. The evaluation considered free versions and the advanced functionalities available in paid versions.

## Results

This study revealed significant benefits of using artificial intelligence (AI) tools in scientific writing. Elicit, Perplexity, and Consensus proved valuable for optimizing literature reviews and scientific analyses. Elicit effectively synthesizes information from multiple articles, generating precise summaries without requiring full readings. Its metadata filters and integration with reference management systems like Zotero and Mendeley streamlined the review process, making it more efficient. However, specific queries produced incomplete or overly simplified results, requiring validation through the sources. Additionally, the free version limited the retrieval of more specialized documents.

Perplexity stood out for its ability to search for updated information and produce concise summaries. Its user-friendly interface facilitated quick navigation between results and contextual citations. Despite these strengths, its responses lacked depth in more complex queries, requiring complementary searches for comprehensive analysis. Consensus, on the other hand, delivered promising results by identifying gaps in the literature and providing evidence-based summaries. Its natural language capabilities simplified query formulation and enhanced the efficiency of literature exploration. The tool also offered valuable support for scientific writing with features such as citation style adjustments and paraphrasing. However, limitations were noted regarding its coverage of peer-reviewed articles, particularly in emerging research areas.

ChatGPT and Grammarly demonstrated strong support in various aspects of scientific writing. ChatGPT effectively generated drafts, synthesized findings, and suggested innovative topics, particularly for introductory and summary sections. However, limitations included generating inaccurate information, or "hallucinations," and references that did not exist, necessitating thorough author review. Grammarly efficiently identified grammatical errors, improved cohesion, and enhanced stylistic consistency. Its plagiarism detection feature, available in the paid version, was particularly helpful during final manuscript revisions. Nonetheless, the tool occasionally struggled with complex scientific terminology, requiring manual intervention to correct specific suggestions.

The evaluation of AI tools highlighted their diverse applications in scientific writing, from literature searches to language refinement. Table 1 provides a structured summary of the key findings regarding the usefulness and limitations of each tool.

**Table 1:** Summary of AI Tools for Scientific Writing: Applications.

AI Tools	Applicability & Usefulness	Limitations
Elicit	Extracts key points from multiple papers, organizes metadata, and integrates with reference managers; useful for literature review.	Summaries can lack nuance, and effectiveness is reduced in the free version due to restricted database access.
Perplexity	Real-time literature search with citations; effective for quick reference gathering	Shallow analytical depth, citations need validation, and limited free access to high-quality sources
Consensus	Summarizes peer-reviewed studies and helps identify research gaps; valuable for systematic reviews	May not cover the latest research due to reliance on indexed peer-reviewed studies; requires critical analysis.
ChatGPT	Drafting content, summarizing research, and generating ideas; useful for structuring scientific writing.	May produce inaccurate or fabricated content (“hallucinations”); requires fact-checking; lacks deep contextual understanding; ethical concerns with undisclosed use.
Grammarly	Grammar, coherence, style refinement, and plagiarism detection enhance clarity and readability.	Limited handling of complex scientific terminology and accuracy depends on the text context.

**Figure 1:**
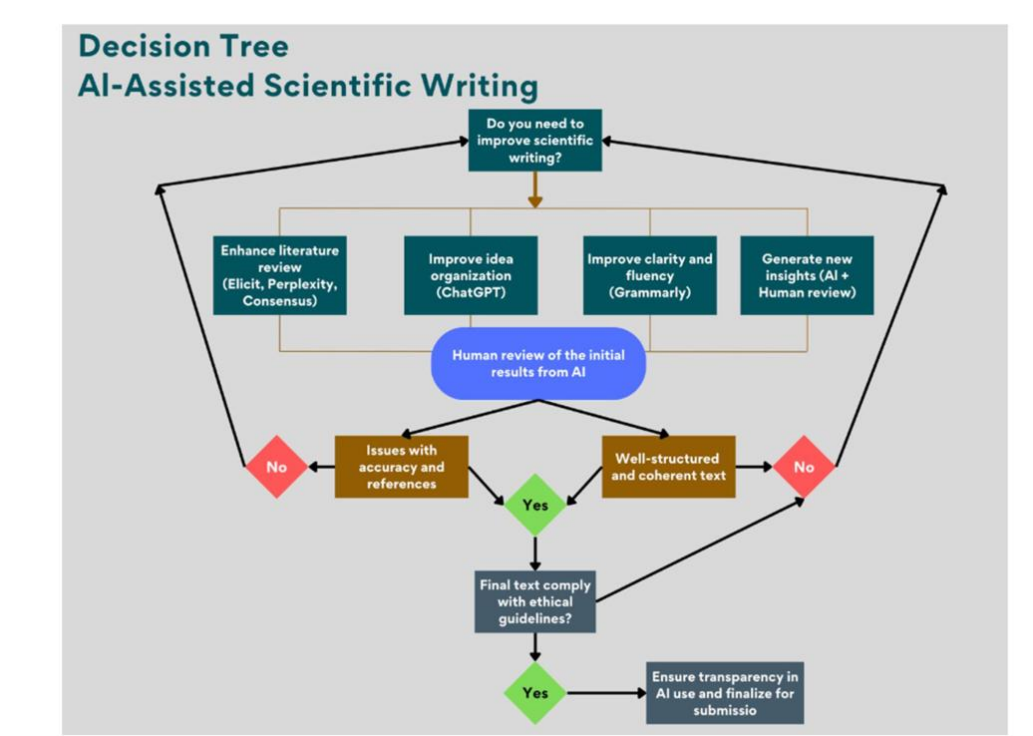
Decision tree for AI-assisted scientific writing. This diagram outlines the workflow for integrating AI tools into scientific writing, from identifying research needs to ensuring compliance with ethical guidelines before submission. The process highlights the role of AI in literature review, text organization, and refinement while emphasizing the necessity of human oversight for quality assurance.

A decision tree was developed to further illustrate the decision-making process of AI-assisted scientific writing (Figure 1). This framework guides researchers in effectively selecting and applying AI tools, considering key aspects such as literature review, text refinement, and ethical compliance. The decision-making path starts by identifying the primary need-improving literature search, structuring ideas, enhancing text clarity, or generating novel insights. AI tools such as Elicit, Perplexity, and Consensus assist in the literature review, while ChatGPT aids in idea organization, and Grammarly improves language accuracy.

After obtaining the initial AI-generated results, researchers must assess the quality of the text. If the output is well-structured and coherent, human review ensures final refinements. However, additional manual adjustments are necessary if issues such as inaccurate references or inconsistencies arise. The final step involves evaluating whether the use of AI complies with ethical guidelines. The manuscript is ready for submission if the AI use aligns with academic integrity standards. Otherwise, researchers should ensure transparency in AI use, make necessary revisions, and finalize the manuscript for submission.

## Discussion

Artificial intelligence (AI) tools in scientific writing have revolutionized processes where precision and clarity are essential for advancing knowledge. Tools such as Perplexity and Consensus have proven excellent allies in literature search and analysis, helping scientists quickly and accurately identify trends and gaps in scientific production. For non-native English-speaking researchers, tools like ChatGPT and Grammarly have become true partners, helping to overcome language barriers and improve text clarity and coherence, making scientific publications more accessible and impactful.

Despite the advancements, the limitations of these tools still pose significant challenges^6^. The standardization of writing style observed in ChatGPT can restrict the creativity and individual expression of authors^9^. Technical issues, such as the generation of inaccurate information (hallucinations) and incorrect references, highlight the need for rigorous human oversight and validation of results^6^. Tools like Elicit, which rely on heuristics such as citation counts, may compromise reliability in literature reviews, primarily when used without critical oversight.

While AI tools like ChatGPT can assist in scientific writing, concerns remain regarding the risk of standardized and formulaic text. However, the formal style of scientific writing relies on fundamental principles such as logic, clarity, and objectivity to effectively communicate research findings.

Scientific writing is designed to convey complex ideas in a clear and concise manner, making research accessible for understanding and evaluation. It prioritizes precision, using straightforward language to minimize ambiguity and limiting technical jargon to what is necessary for the intended audience. Objectivity is essential, ensuring that facts and evidence take precedence over personal opinions or biases.

Typically structured in a logical format, such as IMRAD (Introduction, Methods, Results, and Discussion), scientific writing helps guide readers through the research process. Every claim must be supported by data and credible sources, reinforcing the reliability of the work. Conciseness is also key-unnecessary words and overly complex sentences are avoided to maintain readability.

Although the passive voice is traditionally used to emphasize actions or processes rather than the researcher, writing conventions may vary by discipline. Additionally, proper referencing and citations are crucial for transparency, enabling readers to verify sources. Ultimately, effective scientific writing is essential for disseminating research, advancing knowledge, and fostering meaningful dialogue within the scientific community.

Ethical issues play a central role in the use of AI in scientific writing^10^. Aspects such as the definition of authorship and responsibility for the content generated require clear and applicable guidelines in practice. In Brazil, initiatives like the guide from the Senai Cimatec University Center have emphasized transparency, human centrality, and data privacy protection as fundamental aspects^11^. Recommendations from the Federal University of Minas Gerais (UFMG) further strengthen the debate by highlighting the role of AI literacy in empowering teachers, researchers, and students to use the technology responsibly^12^. These initiatives also warn of risks such as plagiarism, misinformation, and discriminatory biases, which the improper use of AI^13^ may exacerbate.

These Brazilian regulations offer a practical framework adaptable to other academic settings, including initiatives such as ethics courses on AI use and mandatory declarations regarding technology in manuscripts. These measures foster transparency and accountability, encouraging responsible integration of AI tools in research.

A key reference in this context is SciELO's *Guide for the Use of AI Tools in Research Communication*, which provides comprehensive recommendations for authors, editors, and reviewers ^14^. The guide underscores the importance of transparency and ethical compliance in research communication. Authors are expected to disclose any use of AI tools during the preparation, writing, or revision of manuscripts, with details included in both the abstract and methodology sections. This guideline reinforces that authorship is a human responsibility, emphasizing accountability for the integrity of scientific work.

Editors and reviewers are equally tasked with ensuring ethical oversight by monitoring and documenting AI usage throughout the editorial process. This oversight aims to prevent ethical violations, including misinformation and the concealment of AI contributions. The guide also encourages the adoption of tools and training to detect and address AI-generated content, safeguarding the quality and trustworthiness of academic publications.

By providing clear guidance on ethical AI use, SciELO's initiatives contribute to a growing movement that seeks to balance technological advancements with academic integrity. This framework is a practical reference for institutions implementing responsible research and scholarly communication practices.

In science, where precision in describing clinical and laboratory data is essential ^3^, AI emerges as a promising opportunity to enhance the efficiency and quality of scientific publications. However, its adoption should be accompanied by continuous human oversight and adherence to institutional guidelines that ensure academic integrity and the ethical advancement of science.

While a powerful tool, AI should complement human work rather than replace it, there have already been suggestions to use the term "co-intelligence" instead of "artificial intelligence"^15^. Its implementation requires a balance between innovation and responsibility, with specific regulations to maximize its benefits and minimize risks^16^. Continuous supervision and rigorous ethical practices are essential to ensure that AI solidifies as a robust ally in scientific writing, preserving the quality, originality, and integrity of academic contributions while preventing potential issues related to plagiarism ^17^. Recent proposals have emphasized the necessity of structured guidelines for AI use across all stages of scientific publishing. Meneses (2025)^18^ presented a comprehensive framework of responsibilities for authors, reviewers, and editors, reinforcing the importance of transparency in prompt reporting and delineating ethical boundaries for AI use in manuscript preparation and evaluation ^18^.

Although this study focused on evaluating AI tools' current applications, further research is underway to explore how these tools can support the development of article structure and scientific argumentation in greater detail.

## Conclusion

Integrating AI in scientific writing offers significant benefits, including enhanced productivity, improved access for non-native English-speaking researchers, and optimized literature reviews. However, these advantages are accompanied by challenges related to technical accuracy, ethical authorship, and standardized writing styles. We conclude that AI tools can complement human expertise but must be implemented with rigorous ethical oversight and institutional guidelines to maintain the integrity and originality of scientific work.

### Statement on the Use of Artificial Intelligence (AI)

In preparing this manuscript, the authors utilized AI tools to enhance the efficiency and quality of the writing process. Specifically:

- ChatGPT was employed to generate text drafts, refine structure, and synthesize key concepts during the early stages of manuscript development.
- Grammarly was used to improve clarity, grammatical accuracy, and stylistic consistency throughout the manuscript, with special attention to sentence structure and coherence.
- Perplexity supported the research process by providing concise summaries of recent academic literature and contextual citations, aiding in identifying relevant sources.

The use of these AI tools adhered to ethical guidelines for scientific communication, with all content generated by AI reviewed and validated by the authors to ensure accuracy, integrity, and originality. The authors take full responsibility for the final content of the manuscript.
